# Mind the Spike: Compliance With Inpatient Hyperglycaemia Management Guidelines on an Acute Mental Health Ward in Nottinghamshire

**DOI:** 10.1192/bjo.2026.11807

**Published:** 2026-06-30

**Authors:** Oluwabunmi Ogungbemi

**Affiliations:** Nottingham healthcare NHS foundation Trust, Nottingham, United Kingdom

## Abstract

**Aims:**

Hyperglycaemia is common among psychiatric inpatients due to comorbid diabetes, medication side-effects, and challenges in self-care. Poor recognition and escalation increase risk of diabetic emergencies.

There is also the ongoing dilemma of Insulin misuse and poor glucose management, as a form of self-harm in a subset of psychiatry patients. These factors lead to increasing difficulties in managing patients with Diabetes admitted on inpatient wards.

This audit aimed to assess compliance with the Trust’s Inpatient Hyperglycaemia Guideline for episodes of blood glucose >15 mmol/L on an Acute male inpatient Ward in Nottinghamshire.

**Methods:**

- Retrospective audit of RiO electronic records
- Records reviewed over a 6 month period, spanning January–August 2025
- 30 hyperglycaemic episodes (>15 mmol/L) in 7 patients.
- Results collected, organized and analysed using Microsoft excel.
- Standards assessed: ketone testing, wellness status reviews, escalation to medic, clear documentation, insulin use and repeat monitoring.
- Standards were devised based on the Nottinghamshire Healthcare NHS Trust’s “Inpatient guide for acute Hyperglycaemia in Diabetes Type 1 or 2”,

**Results:**

- Ketones checked in**14/30 (46.7%)**
- Wellness status documented in **14/30 (46.7%)**, NEWS2 recorded only **3/30(10%)**
- Escalation gaps: **7/25(23.1%) episodes with BG >18 mmol/L not escalated to a medic.**
- No documentation of pre-meal glucose review in **all cases.**
- Insulin dosing appropriate in **36.7% of cases with an indication.**
- BG rechecked at 2h in **10/30(33.3%)**, at 4h in **7/30 (23.3%)**
- Cause identified in **10/30 (33.3%)**, mostly dietary

**Conclusion:**

Compliance with inpatient hyperglycaemia management standards was variable, with significant gaps identified in ketone assessment, clinical monitoring, documentation of pre-meal trends, documentation of wellness status and escalation practices. These findings highlight the ongoing challenges of delivering consistent physical healthcare within mental health inpatient settings. Targeted system-level interventions, including staff education and structured patient-centred care planning, may improve adherence to guidelines and reduce risk of preventable metabolic complications.

This has led to ongoing collaborative plans to develop an individualised diabetes care plan template, along with improved referral systems to Diabetes specialist nurses.

